# Oncocytic and Follicular Thyroid Carcinomas Arising in Hashimoto’s Thyroiditis: Two Rare Cases Highlighting an Unusual Association

**DOI:** 10.7759/cureus.114471

**Published:** 2026-08-13

**Authors:** Nicholas Cassar, Miljan Milic

**Affiliations:** 1 Cellular Pathology Section, Department of Pathology, Mater Dei Hospital, Msida, MLT

**Keywords:** follicular carcinoma of the thyroid, hurthle cell carcinoma, oncocytic thyroid carcinoma, papillary carcinoma of thyroid, hashimoto’s thyroiditis

## Abstract

Hashimoto’s thyroiditis (HT) is strongly associated with papillary thyroid carcinoma (PTC). On the other hand, well-differentiated non-PTC thyroid follicular cell-derived malignancies are exceedingly uncommon in a HT milieu. We report two such cases.

Two female patients (aged 36 and 23 years) each presented with a thyroid nodule. In both patients, histopathologic assessment confirmed nPTC - one a minimally invasive oncocytic carcinoma (capsular invasion only, pT2) and the other a conventional minimally invasive follicular carcinoma (capsular invasion only, pT3a) - arising on a background of florid chronic lymphocytic thyroiditis and raised anti-thyroid peroxidase antibodies. No features of papillary differentiation or angioinvasion were identified. Both were marginally completely excised, and subsequent completion thyroidectomy specimens showed no residual malignancy.

While HT is a known risk factor for PTC, it may also - albeit rarely - be associated with other well-differentiated thyroid follicular cell-derived malignancies. These two cases exemplify rare occurrences of minimally invasive oncocytic and follicular thyroid carcinomas arising in florid HT. Their recognition is essential to avoid misclassification and continues to challenge assumptions regarding malignancy associated with autoimmune thyroiditis, raising questions about the molecular mechanisms at play.

## Introduction

Hashimoto’s thyroiditis (HT) is a common autoimmune condition with a worldwide prevalence of 5%-10% in adults [1,2]. It has been linked to papillary thyroid carcinoma (PTC) in numerous studies [3-5], possibly due to *RET/PTC * pathway activation in HT [6-9].

In contrast, the coexistence of HT with well-differentiated non-PTC thyroid follicular cell-derived malignancies (nPTCs) such as oncocytic (OTC) and follicular (FTC) thyroid carcinomas is unusual and comparatively underreported - particularly so for OTC, with fewer than five cases reported in the English literature [10-14]. We report two cases of nPTC in HT.

## Case presentation

Case 1

**Figure 1:**
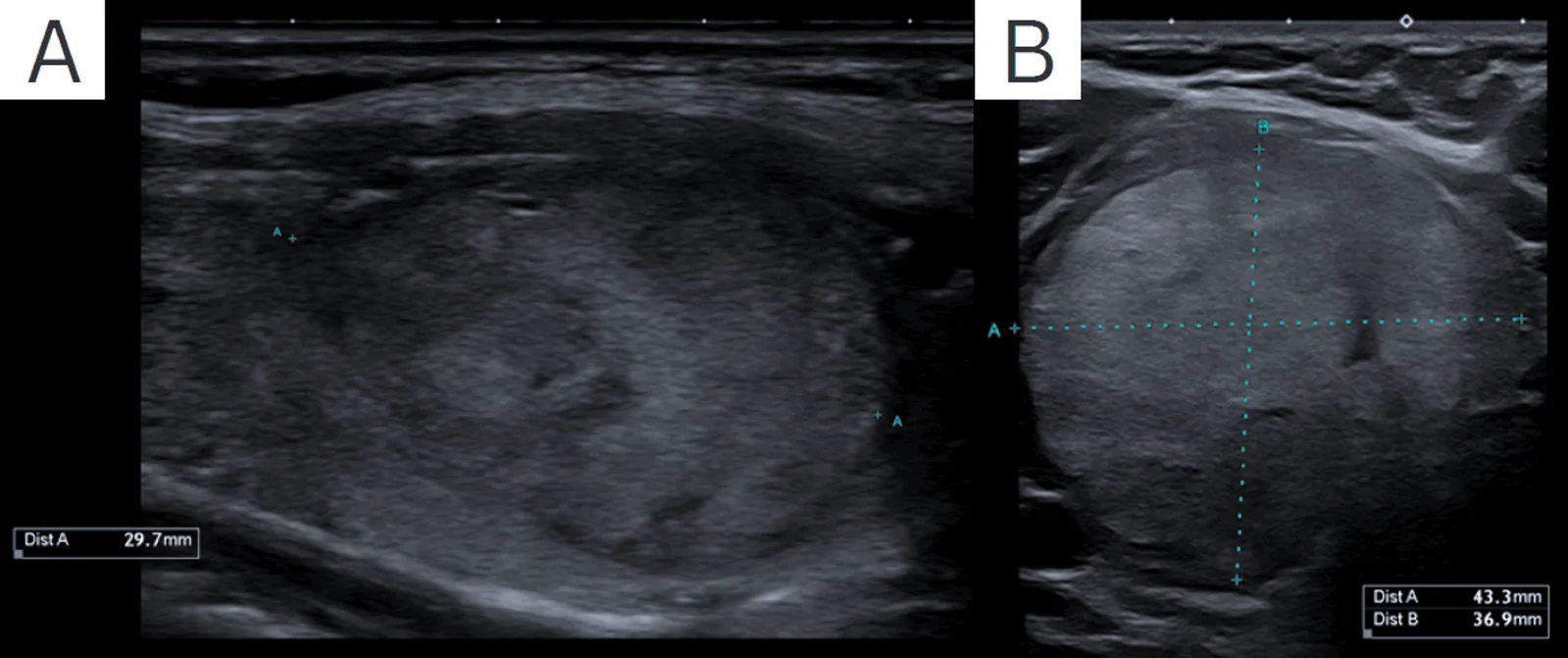
Ultrasound images showing greatest radiological dimension of nodules of first (A) and second (B) cases

A 36-year-old woman presented with a 3 cm×2. 2 cm×1.8 cm left thyroid nodule, classed as American College of Radiology Thyroid Imaging Reporting and Data Systems (ACR-TIRADS) category 3 and as Bethesda System for Reporting Thyroid Cytopathology (BTC) category 3 on ultrasound (Figure 1A) and cytopathological assessment, respectively. The patient underwent a left hemithyroidectomy. Gross examination of the resection specimen revealed a well-circumscribed nodule with a tan solid and haemorrhagic cut surface, measuring 24 mm in maximum dimension. This was embedded in its entirety.

**Figure 2:**
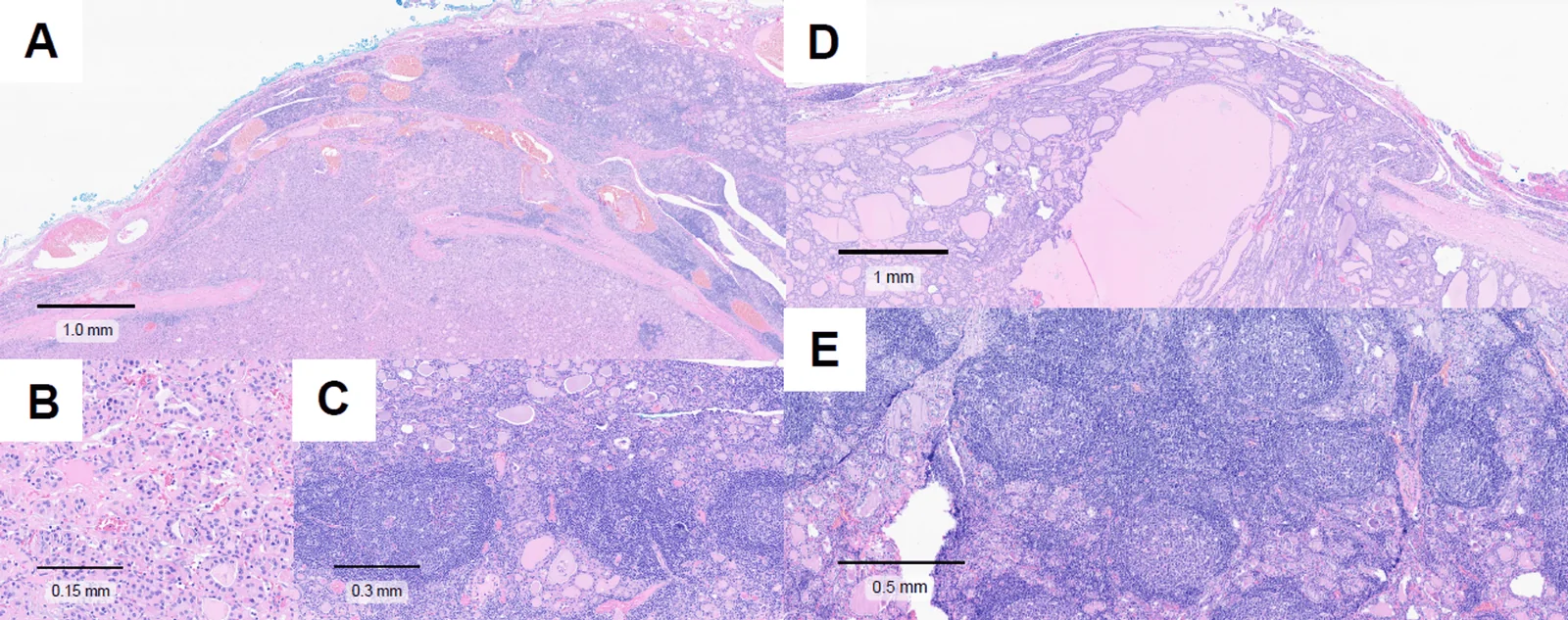
Haematoxylin and eosin-stained light microscopy images of first (A-C) and second (D, E) cases A. Full-thickness capsular invasion, first case B. Oncocytic cells with microfollicular pattern, first case C. Background thyroid parenchyma involved by lymphoid aggregates with frequent germinal centre formation typical of HT, first case D. Full-thickness capsular invasion, second case E. Background thyroid parenchyma involved by lymphoid aggregates with frequent germinal centre formation typical of HT, second case

Microscopically, the nodule consisted of an encapsulated tumour composed of microfollicles lined by oncocytic cells (Figure 2B) without papillary nuclear features and without significant nuclear atypia nor other high-grade features. Multiple foci of complete capsular penetration were present (Figure 2A), but no angioinvasion was noted. The lesion was marginally completely excised. Immunohistochemistry showed strong diffuse thyroid transcription factor 1 (TTF-1) expression, weak but diffuse thyroglobulin expression, and negative synaptophysin and calcitonin expression, confirming thyroid follicular epithelial differentiation. The background thyroid parenchyma exhibited florid chronic lymphocytic thyroiditis with germinal centre formation and oncocytic metaplasia (Figure 2C). Given that anti-thyroid peroxidase antibody levels taken previously were elevated to more than 10 times the upper limit of normal (>500 IU/ml), the background histological findings were most in keeping with HT.

The diagnosis was minimally invasive oncocytic thyroid carcinoma (OTC), pT2, on a background of HT. Completion right hemithyroidectomy showed lymphocytic thyroiditis only, with no residual carcinoma.

Case 2

A 23-year-old woman presented with a 4.3 cm×3.7 cm right isthmic nodule, classed as ACR-TIRADS 3 and as BTC 2 on ultrasound (Figure 1B) and cytopathological assessment, respectively. The patient underwent right hemithyroidectomy with isthmusectomy. Gross examination of the resection specimen revealed a well-circumscribed, encapsulated tan-brown solid lesion measuring 49 mm in maximum dimension. This was sampled extensively.

Microscopically, the nodule consisted of an encapsulated tumour comprised of variably sized follicles lined by thyroid follicular epithelial cells without papillary nuclear features and without significant nuclear atypia nor other high-grade features. Multiple foci of complete capsular penetration were present (Figure 2D), but no angioinvasion was noted. The lesion was marginally completely excised. The background thyroid parenchyma exhibited florid chronic lymphocytic thyroiditis with germinal centre formation and oncocytic metaplasia (Figure 2E), in keeping with HT, corroborated by pre-operative anti-thyroid peroxidase antibody levels elevated to seven times the upper limit of normal (351 IU/ml). 

The diagnosis was minimally invasive follicular thyroid carcinoma (FTC), pT3a, on a background of florid chronic lymphocytic thyroiditis. Completion left hemithyroidectomy with left level VI lymph node dissection showed lymphocytic thyroiditis only, with no residual carcinoma nor metastasis (0/11 lymph nodes).

## Discussion

Although HT and PTC share a well-established association, that between HT and nPTCs is rare. This compounds the already significant diagnostic limitation of fine-needle aspiration cytology (FNAC), which cannot reliably distinguish between follicular or oncocytic thyroid adenomas versus invasive carcinomas [15]. Oncocytic metaplasia is also a hallmark of HT typically noted on FNAC [12,16,17], yet true OTC developing in this milieu is vanishingly rare. Histological confirmation of true capsular and/or vascular invasion - as demonstrated in both the above-reported cases - is essential for the diagnosis of invasive thyroid carcinoma as per World Health Organization 2022 criteria [15].

Of note, although the prevalence of PTC is increased in HT, PTC in HT generally has a more favourable prognosis than in patients without HT, although defining the molecular mechanisms underpinning these phenomena with certainty remains challenging [9].

As mentioned previously, it has been posited that HT and PTC share a pathophysiological connection through RET/PTC activation. On the other hand, both the predominantly *RAS*-driven FTC and the more molecularly heterogeneous OTC are biologically distinct from the predominantly *BRAF* V600E or *RET/PTC*-driven PTC [15] - and yet they have still been reported in HT, albeit far less frequently.

It remains to be seen whether the rarity of these associations is due to protective mechanisms, under-reporting, or confounding or other factors and whether the cases of nPTCs that do arise in HT behave differently - and if so, in what way and why - to nPTCs in patients without HT.

Given the complexity of the possible immunological mechanisms at play, the myriad potential protagonists and supporting molecular targets, and the relative rarity of FTC and (especially) OTC compared to PTC, it seems the discussion would best be settled by large-scale multi-center studies with generous use of molecular investigative techniques.

Pending such studies, what these and similar case reports do show is that a diagnosis of HT does not and should not ipso facto exclude a diagnosis of nPTC, and vice versa.

## Conclusions

While HT is a known risk factor for PTC, it may also - albeit rarely - be associated with other well-differentiated thyroid follicular cell-derived malignancies. These two cases exemplify rare occurrences of minimally invasive oncocytic and follicular thyroid carcinomas arising in florid Hashimoto’s thyroiditis. Their recognition is essential to avoid misclassification and continues to challenge assumptions regarding malignancy associated with autoimmune thyroiditis, raising questions about the molecular mechanisms at play.
